# Polyamory

**DOI:** 10.1192/j.eurpsy.2022.2259

**Published:** 2022-09-01

**Authors:** R. Cavalli, G. Rogier, P. Velotti

**Affiliations:** 1 University of Rome Sapienza, Dynamic And Clinical Psychology And Health, Rome, Italy; 2 University of Genoa, Department Of Educational Sciences, Genoa, Italy

**Keywords:** Polyamory, attachment, Emotion dysregulation

## Abstract

**Introduction:**

Few studies investigated the role of psychological variables underlying polylove.

**Objectives:**

To extend the knowledge regarding the psychological profile of polylovers.

**Methods:**

We administered to a sample of individuals reporting to be polylovers and a sample of participants reporting to not be polylovers a battery of self-report questionnaires including the Attitude Towards Polylove scale (ATP), the Multidimensional Sexuality Questionnaire (MSQ), The Experiences in Close Relationships 12 items (ECR-12), the Couple Satisfaction Inventory (CSI), the Difficulties in Emotion Regulation Scale (DERS) and the Difficulties in Emotion Regulation Scale Positive (DERS-P).

**Results:**

We found that controlling for age and gender, polylovers, compared to not polylovers, scored higher on some dimensions of the DERS-P, on the ATP scores and on some dimensions of the MSQ. No others significant differences between groups emerged. Moreover, in the group of polylovers, ATP scores were positively related to sexual satisfaction, sexual self-esteem and sexual consciousness and negatively related to avoidant attachment style and difficulties in regulating positive emotions. Finally, we found that avoidant attachment style moderated the link between ATP scores and sexual self-esteem.

**Conclusions:**

Emotion dysregulation and attachment appear to be central variables explaining the specificity of psychological profiles of polylovers.

**Disclosure:**

No significant relationships.

